# Perceptions of Digital Health App Usage Among Women Attending Obstetrics and Gynecology Outpatient Department in a Tertiary Care Setting

**DOI:** 10.7759/cureus.82605

**Published:** 2025-04-19

**Authors:** Amrita Datta, Jaya Shankar Kaushik, Himangshu Malakar

**Affiliations:** 1 Obstetrics and Gynaecology, All India Institute of Medical Sciences, Guwahati, Guwahati, IND; 2 Paediatrics, All India Institute of Medical Sciences, Guwahati, Guwahati, IND

**Keywords:** abha (ayushman bharat health account), digital health, patient perception, tertiary care, women's health

## Abstract

Introduction

Digitalization is transforming healthcare, with tools like digital health apps and platforms becoming integral. India's Ayushman Bharat Digital Mission (ABDM) aims to create a digitally empowered healthcare system, utilizing the Ayushman Bharat Health Account (ABHA) for seamless access to health services. This study evaluates patients' perceptions of digital healthcare services, specifically ABHA, in a tertiary care setting, and identifies factors influencing adoption.

Methods

A cross-sectional study was conducted over six months (September 2024-March 2025) among 250 women (aged 18-80) attending the Obstetrics and Gynecology outpatient department (OPD). A validated questionnaire assessed patient perceptions of ABHA registration and digital health records. Demographic data and awareness of digital platforms were collected. Statistical analysis included descriptive statistics, chi-square tests, and logistic regression.

Results

The mean age of participants was 28 ± 3.2 years. Awareness of digital platforms varied, with 125 (60%) being aware of digital India platforms, 87 (35%) of ABHA, and 50 (20%) of the Swasthya app. The majority of participants (100 (40%)) belonged to the upper lower class of the Kuppuswamy socioeconomic scale. Overall, patients reported high satisfaction with ABHA registration (satisfaction index (SI) = 87.9), perceiving it as simple and efficient. Logistic regression showed no significant influence of age, education, socioeconomic status, or ABHA awareness on digital health app usage. However, the scan and share feature had a lower SI (78.7).

Discussion

While patients generally viewed ABHA positively, awareness gaps and usability challenges, particularly with the scan and share feature, were identified. Socioeconomic factors did not significantly impact adoption, suggesting effective integration of ABDM into public healthcare. Targeted educational programs and technological improvements are necessary to enhance digital health literacy and adoption.

Conclusion

This study highlights the importance of addressing awareness and usability barriers to optimize digital health adoption in tertiary care settings. Enhancing patient education and improving digital interfaces can improve healthcare delivery through digital platforms.

## Introduction

Digitalization is the present way of life, and healthcare is one of the sectors where its presence is well felt. Digitalization of health services, including telemedicine, has made healthcare easily accessible to a wider group of people [1]. Reports have suggested increased patient uptake and better utilization of healthcare resources through digitalization [1,2]. Newer digital tools, such as healthcare applications and online patient portals, have helped both patients and healthcare professionals by providing ease of usage and wide access to clinical data [2]. With an aim to create a digitally empowered society and to ensure digital health for all, India launched the National Digital Health Mission in 2021, now known as Ayushman Bharat Digital Mission (ABDM) [3,4]. A pivotal part of the program is the creation of the ABHA or the Ayushman Bharat Health Account number [4]. This is a unique 14-digit ID that allows individuals to access healthcare services nationwide and link their health benefits, like insurance, to their ABHA record [4].

In hospitals equipped with the ABDM facility, patients can digitally register using the ABHA app or other compatible platforms like the Driefcase app on their smartphones. After registration, the ABHA ID can be linked to the healthcare facility, which is common to any ABDM-enabled system, through individualized barcodes. This integration enables seamless access to laboratory reports, digital prescriptions, and appointment scheduling from anywhere through ABDM-enabled gateways [4]. This has been shown to provide better experiences to the patients and their providers alike [4]. Moreover, the advent of pandemics like COVID has seen the adoption of digital health tools like ABHA with increasing rapidity [5]. Despite digital health tools being designed for patients' perceived needs, there is limited data on the perspectives of patients regarding their usage [2,6].

With the hypothesis that patients at a tertiary healthcare facility perceive digital health services, including ABHA and related tools, as beneficial and user-friendly, this study primarily aimed to assess their perspectives and experiences regarding digital healthcare tools. Secondary objectives included identifying factors influencing their perception and adoption of digital health applications.

## Materials and methods

Methodology

Study Design and Study Population

This cross-sectional study was conducted for six months from September 2024 to March 2025. The study enrolled all adults aged 18-80 who visited the Obstetrics and Gynecology outpatient department (OBG OPD) for consultation during that period through a convenience sampling approach. Individuals with less than a fifth-grade education and those unable to comprehend either English or the local language were excluded. Additionally, participants who consented but provided incomplete questionnaires were also excluded.

With a frequency of usage of digital tools of 76% and a confidence interval of 95%, the sample size was calculated to be 250 based on a reference study looking at digital app usage in healthcare [7]. The power and precision were adjusted to be 80% and 6%, respectively.

Written informed consent was obtained from all eligible subjects, and institutional ethical clearance was secured before the study commenced (ref. no. M7/F205/2024).

Study Setting

The study was conducted at a tertiary care institute in Northeast India, catering to patients from Assam and other northeastern states, and has implemented the Ayushman Bharat Digital Health Mission. In the facility, patients are encouraged to generate an ABHA number at the outpatient entry. For those without smartphones, ABHA is generated at the registration counters by the hospital volunteers.

The ABHA is then linked to the Hospital Management Information System (HMIS) via a “scan and share” and a token number unique to each patient created after the initial registration. The token number is then submitted at ABHA registration counters to receive OPD slips for doctor consultations. Once the consultation is completed, doctors upload the prescription slips online. The laboratory tests, which are also ordered online, are processed in the designated area, and results are uploaded to the mobile app thereafter. Patients can access their online prescriptions and laboratory reports through the eSwasthya App developed under HMIS.

Development and Validation of the Study Questionnaire

A study questionnaire was designed and validated to assess patients' perceptions of ABHA registration and the digitalization of health records. Initially, the investigators held focused group discussions involving healthcare professionals and ABHA volunteers. Insights from these discussions helped shape the questionnaire's content by identifying key aspects of ABHA registration. The final version was designed with the target audience in mind and made easy to understand. The reliability of the questionnaire was also checked by administering it to the same participants at different time points of the study to ensure test-retest validity.

The drafted questionnaire was reviewed by both patients and healthcare professionals for clarity and relevance. It was translated into the local language and underwent expert validation with back translation. Subsequently, it was pilot tested with 10 participants. Based on the feedback, the study questionnaire was finalized after necessary modifications. The final version included 10 questions rated on a five-point Likert scale, along with several closed-ended questions about ABHA registration (Appendices). Patients were guided by nursing officers in the OPD in case of queries regarding the questions. Likert values were considered ordinal while analyzing the final responses. Satisfaction index (SI) was calculated by summating the number of subject responses of four and five (in the Likert scale) and dividing it by the total number of responses (scores 1 to 5), which was rounded off to a percentage.

Demographic parameters such as age, gender, educational qualifications, socioeconomic status, and occupational status were also recorded.

Statistical Analysis

Data were entered in Microsoft Excel and analyzed using SPSS (IBM SPSS Statistics for Windows, IBM Corp., Version 22.0, Armonk, NY). Categorical variables were presented as numbers (proportions), while continuous variables were expressed as mean (SD) or median (IQR). Logistic regression analysis was used to determine factors influencing patients' responses to ABHA. The responses were analyzed in relation to the socio-demographic parameters using the chi-square test. A value of less than 0.05 was considered significant. Participants with any of the missing data parameters were excluded from the final calculation.

## Results

A total of 250 patients attending the OPD during the study period participated in the study. The mean age of our patients was 28 ± 3.2(mean ± SD). The majority (156 (60%)) were first-time visitors. The study had 100 (40%) patients presenting for antenatal care. The demographic details are summarized in Table 1.

**Table 1:** Demographic details of enrolled patients (n = 250)

Variable		n (%)
Locality	Rural	175 (70%)
	Semiurban	50 (20%)
	Urban	25 (10%)
Socioeconomic status (Kuppuswamy class)	Upper	7 (3%)
	Upper middle	20 (8%)
	Lower middle	70 (28%)
	Upper lower	100 (40%)
	Lower	53 (21%)
Educational status	Graduate	100 (40%)
	High school	85(24%)
	Middle school	25(10%)
	Primary school	20 (8%)
	Illiterate	20 (8%)
Occupation	Homemaker	193 (77%)
	Professional	53 (23%)

Among the study participants, 125 (60%) were aware of digital India platforms, 87 (35%) had knowledge of ABHA, while around 50 (20%) were familiar with the Swasthya mobile app. Logistic regression analysis revealed that none of the examined variables, including age, educational status, socioeconomic status based on the Kuppuswamy classification, awareness of ABHA, or revisit status, were significant in influencing digital app usage (Table 2).

**Table 2:** Logistic regression analysis table comparing variables with digital app usage

Variable	OR	95% CI	p-value	Nagelkerke R^2^ values
Age	1.15	0.84-1.38	0.75	-
Educational status	0.84	0.72-1.17	0.81	-
Socioeconomic status (Kuppuswamy class)	0.71	0.6-1.21	0.8	-
Awareness of ABHA	0.51	0.46-1.01	0.06	-
Revisit status	0.91	0.31-2.68	0.21	-
Model Nagelkerke R^2^	-	-	-	0.071

Overall, patients were satisfied with the ABHA registration process (SI = 87.9) and considered it simple and required no additional assistance (SI = 86.1). Patient responses are depicted in Table 3.

**Table 3:** Satisfaction index (SI) of the experience of ABHA

Variables	1 (strongly disagree)	2 (disagree)	3 (neutral)	4 (agree)	5 (strongly agree)	Number responded	SI
Experience with ABHA registration	1	3	25	62	149	240	87.9
Experience regarding saving OPD queue time through ABHA registration	1	6	20	60	153	240	88.7
Simple process requiring little to no assistance	3	10	20	60	145	238	86.1
Easy-to-use scan and share options in ABHA	2	13	35	50	135	235	78.7
Ease of usage of ABHA as perceived by antenatal patients	2	10	25	60	138	235	84.2
Ease of usage as perceived by patients presenting with gynecological complaints	3	8	15	70	144	240	89.1

## Discussion

The digitalization of healthcare services is still in the nascent phase in India [4]. The National Digital Health Mission and its pilot program, ABDM, aspire to streamline healthcare services in our country by empowering patients, doctors, and healthcare facilities [4,8]. The present study evaluated the perception and experience of digital health application usage among OBG OPD attendees in a tertiary care setting.

Perception and awareness of digital health platforms

Digital health tools aim to enhance healthcare accessibility and efficiency; however, patient awareness remains a crucial determinant of their adoption. The study revealed that 125 (50%) participants were aware of digital India platforms (including ABHA), while only 87 (35%) had specific knowledge of ABHA. These findings align with previous studies indicating that although digital health initiatives are widely promoted, patient awareness and usage remain limited, particularly in rural settings [8]. A key observation in this study was that awareness of ABHA was not significantly influenced by age, educational status, or professional status (p > 0.05). This might suggest that ABHA and other digital tools are convenient for everyone to use and could make digital healthcare inclusive. This is in contrast to reports that identified poor uptake among those with low educational status and increasing age [7]. Targeted awareness programs may still be needed to bridge the knowledge gap across diverse demographic groups [9,10].

User experience and satisfaction

The study found an overall positive patient perception of the ABHA registration process at a SI of 87.9. Most participants found it easy to generate ABHA. Additionally, the efficiency of ABHA in reducing OPD queue time was well-received (SI = 88.7). These findings support previous literature indicating that digitalization of healthcare can improve patient experience and streamline clinical workflows [8,11]. However, in this study, the SI of antenatal patients was found to be poor in comparison with those who came for gynecological problems. This could be due to the fact that pregnant patients were preoccupied and had less time to think about the ABHA procedure.

The scan and share feature of ABHA, which allows seamless access to digital health records, had a slightly lower SI (78.7). This could indicate challenges in usability, possibly due to some patients' unfamiliarity with digital interfaces or technological barriers [11,12].

Role of socioeconomic factors

Interestingly, socioeconomic status did not significantly influence patient perception or adoption of digital health tools (p > 0.05). This is in contrast to some studies that suggest higher adoption rates among individuals from higher socioeconomic backgrounds, likely due to better access to digital resources [13]. The absence of such an association in this study could be attributed to the government’s effort to integrate ABDM with the existing public healthcare infrastructure, ensuring its accessibility to lower-income groups. Moreover, ABHA can be generated even if the patient does not have access to a smartphone.

Barriers to digital health adoption

Despite the positive perception, the study highlights several barriers to the widespread adoption of digital health apps. One primary concern is the relatively low awareness of ABHA, indicating gaps in patient education and outreach efforts. This finding is consistent with other research suggesting that the success of digital health interventions depends on effective patient engagement strategies [14]. Another potential challenge is digital literacy, which could affect the ease of using a health app [12,14]. Given that 18% of the participants had only a primary-level education or were illiterate, digital literacy initiatives tailored to such groups might be necessary for equitable access to digital health services. Infrastructure limitations also pose a challenge, particularly in rural areas where internet connectivity and smartphone access may be inadequate [14]. Also, several patients expressed concern about the security aspect of ABHA, such as data being linked to AADHAR and chances of data breach in accordance with prior reports [12,14,15].

This study had several limitations. Apart from the small sample size, it looked at patients attending only one department in the OPD setting. A multicentric approach involving various departments with larger sample sizes, particularly in emergency settings, would provide further insights. There is also the possibility of response bias as healthcare staff had helped in the data collection of some of the participants. This could have impacted patient satisfaction and their self-reported use of digital platforms. There might also be chances of social responsibility bias, as some patients would have responded more favorably than others. Digitally literate patients would also have a better response.

Encouraging the routine use of digital health tools for appointment scheduling, accessing test results, and teleconsultations can reinforce their importance and utility among patients, as reported [16]. Digital health education programs could be implemented at the community level, especially for those with limited educational backgrounds, to increase awareness about ABHA and related digital services [12,17]. Training healthcare professionals to assist patients with ABHA registration and app usage could improve overall acceptance [18] and could be considered when framing policies for implementation.

## Conclusions

This study provides valuable insights into the perception and experience of women using digital health applications in a tertiary care setting. While the overall satisfaction with ABHA was high, awareness and usability issues remain key challenges. Addressing these barriers through targeted educational initiatives, technological improvements, and policy interventions can enhance digital health adoption and optimize healthcare delivery.

## Appendices

Questionnaire

Name

Age

Educational status

Monthly family income

Tell us about your locality.

   Rural

   Suburban

   Urban

Tell us about your profession.

   Salaried

   Homemaker

Are you aware of the Digital India Program initiated by the Government of India?

   Yes

   No

   I have just heard the name.

Were you aware of ABHA registration initiated by the Government of India before coming to AIIMS Guwahati?

   Yes

   No

   I have just heard the name.

What is your visit to AIIMS?

   First visit

   Revisit

Were you aware of the AIIMS Guwahati Swasthya App before coming to AIIMS Guwahati?

   Yes

   No

   I have just heard the name.

Please answer the following questions about ABHA registration.

**Table 4:** A few questions on the experience of ABHA registration ABHA, Ayushman Bharat Health Account; AIIMS, All India Institute of Medical Sciences; OTP, one-time password

Questions	Strongly agree	Agree	Neutral	Disagree	Strongly disagree
My experience with ABHA registration was satisfactory.					
Registering the patient through ABHA saved time waiting at the counters.					
Registering through ABHA was simple, and I did not require any guidance.					
I faced problems in registering through ABHA.					
Staff at AIIMS Guwahati helped in creating the ABHA number.					
My experience with the use of scan and share options was satisfactory.					
I had trouble generating the OTP for ABHA registration.					
I was hesitant to share my AADHAR details for fear of my personal details being misused by any third party.					
I understood the benefits of ABHA registration.					
I would like to volunteer and help others with ABHA registration.					
